# Cardiovascular risk profile with SCORE2 and SCORE2-OP: comparing Portugal, Spain, Italy, and France using the new European predictive models

**DOI:** 10.3389/fcvm.2024.1509240

**Published:** 2024-12-18

**Authors:** Mariana Fontainhas, Cristina Gavina, Joana Miranda, Raquel Pereira-Silva, João Guichard, Daniela Seixas, Francisco Araújo

**Affiliations:** ^1^ Tonic App, Porto, Portugal; ^2^ Unidade Local de Saúde de Matosinhos, Senhora da Hora, Portugal; ^3^ Departamento de Medicina, Faculdade de Medicina da Universidade do Porto, Porto, Portugal; ^4^ Departamento Medicina Interna, Hospital Lusíadas Lisboa, Lisboa, Portugal; ^5^ Clínica Universitária, Faculdade de Medicina da Universidade de Lisboa, Lisboa, Portugal

**Keywords:** cardiovascular diseases, Europe, geriatrics, cardiovascular risk factors, SCORE2, SCORE2-OP

## Abstract

**Introduction:**

This study aims to characterize the cardiovascular risk profile in countries with low-to-moderate cardiovascular mortality risk (Italy, Portugal, France, and Spain) using the SCORE2 and SCORE2-OP models. It also examines regional variations and the involvement of healthcare professionals in performing risk assessments.

**Methods:**

A retrospective observational study was conducted using data from 24,434 cardiovascular risk assessments performed between December 2022 and July 2023 through a digital application used by physicians. The assessments used the SCORE2 model for individuals aged 40–69 and the SCORE2-OP model for those aged 70 and older. Risk stratification into “low-to-moderate,” “high,” and “very high” categories was analyzed based on individual risk factors such as age, smoking habits, systolic blood pressure, and cholesterol levels.

**Results:**

Approximately 50%–60% of individuals in these countries were classified as having “high” or “very high” cardiovascular risk. The highest proportions were observed in Portugal (62.44%) and Italy (64.05%), while lower proportions were found in Spain (46.65%) and France (52.74%). Regional analysis identified areas with the highest cardiovascular risk, such as Portalegre in Portugal and Apulia in Italy. Key risk factors included older age, smoking, high systolic blood pressure, and high non-HDL cholesterol. General practitioners were the primary healthcare professionals conducting these assessments.

**Discussion:**

The study highlights a significant proportion of individuals with “high” or “very high” cardiovascular risk in countries with low-to-moderate mortality risk. These findings underscore the need for targeted cardiovascular disease prevention strategies and the crucial role of general practitioners in managing cardiovascular risk.

## 1 Introduction

Cardiovascular diseases are the leading cause of mortality and morbidity in Europe, making their prevention a public health priority (1, 2). Over the past three decades, the cornerstone of prevention has been cardiovascular risk assessment using predictive models that estimate individual risk over a long-term horizon, typically ten years, to reduce it through individualised strategies according to baseline risk (3–6). Published in 2003, the Systematic Coronary Risk Evaluation (SCORE) model, in particular, was promptly adopted and recommended by the European Society of Cardiology for cardiovascular risk stratification (5–7). In 2021, the model was updated—SCORE2—to incorporate more recent epidemiological data and overcome some of the major limitations of the classic SCORE, such as underestimating the total burden of cardiovascular disease by ignoring non-fatal events (8). Additionally, a specific model for people aged 70 years or older (SCORE2-OP) was developed to address the gaps in traditional risk prediction models in older individuals, including the tendency to overestimate risk and the potential benefit of risk reduction (9). Thus, according to the European Society of Cardiology, these two profiles are defined by new thresholds for risk stratification as “low-to-moderate”, “high”, and “very high”, according to age (<50, 50–69, and ≥70 years).

Unfortunately, two years after the adoption of these recommendations, there are still few publications that characterise real-world reclassification of cardiovascular risk profile, which mostly (1) refer to populations from countries classified as high or very high risk in terms of standardised cardiovascular mortality (10, 11); or (2) derive from a limited number of cardiovascular risk assessments in regions with moderate cardiovascular mortality risk (12). Thus, the current knowledge on risk profiles in regions with low or moderate cardiovascular mortality risk remains largely unknown and undervalued (13) limiting the understating of the most prevalent risk factors in such countries and hindering the implementation of more targeted measures to mitigate cardiovascular disease and reduce the associated mortality.

With the aim of characterising the real-world cardiovascular risk profile obtained with SCORE2 and SCORE2-OP in countries with moderate and low cardiovascular mortality risk, we conducted a study based on cardiovascular risk assessments performed on a digital application used exclusively by physicians, in individuals from two moderate risk countries, Italy and Portugal, and two low risk countries, France and Spain.

## 2 Materials and methods

A retrospective observational study was conducted using the assessments performed with a cardiovascular risk calculation tool using SCORE2 and SCORE2-OP models at digital application for physicians' use. The tool (Tonic App) is a CE-marked medical device that provides access to clinical algorithms, decision trees, drug information and calculators. Registered users must provide accurate and verifiable information (e.g., name, professional license number, medical speciality). For integrity measures, this information is subjected to verification. By agreeing with the applications' privacy policy, users consent that aggregated or de-identified data might be used for research purposes, but no personal identifying information can be processed, of either the physicians or their patients. respecting the terms of use and privacy policy of the application (14, 15) and the EU General Data Protection Regulation (Regulation EU 2016/679).

We analysed all cardiovascular risk assessments consecutively performed by physicians, regardless of their speciality, between December 2022 and July 2023. Only assessments from countries where Tonic App is available were included in this study. Thus, we included only assessments from Italy and Portugal (countries with moderate risk of cardiovascular mortality according to the classification of the European Society of Cardiology) or France and Spain (countries with low risk) (1). For each assessment performed with the SCORE2 and SCORE2-OP models, the corresponding cardiovascular risk estimates were processed. Only relevant epidemiological and clinical data were collected, and data were identified by a code corresponding each subject's data set. This data consisted of the age in years of the individuals who had their cardiovascular risk assessed, their smoking habits (“yes/no”), their systolic blood pressure in mmHg, and their serum levels of total cholesterol and high-density lipoprotein (HDL) cholesterol in mg/dl or mmol/L, as well as their country of origin (Italy, France, Portugal or Spain). The risk estimates were calculated using the SCORE2 model for assessments in individuals aged 40–69 years and with the SCORE2-OP model for assessments in individuals aged 70 years or older (8, 9). The risk stratification was estimated according to the thresholds defined in the 2021 recommendations of the European Society of Cardiology, in the classes of “low-to-moderate”, “high”, and “very high” cardiovascular risk, based on age groups (<50, 50–69, and ≥70 years), for each of the risk estimates obtained (1).

These risk estimates and categories were analysed separately for each country (Italy, France, Portugal and Spain), risk factors recorded from December onwards (7-month period) and sub-analysis of risk classes during that period. The comparison of the four groups was performed using descriptive and inferential statistics, assuming the cardiovascular risk assessments were a subset of a theoretical population consisting of all the assessments that these doctors could perform on eligible individuals from these four countries. The individual risk factors were also described separately for each cardiovascular risk class, as well as for age groups younger than 70 years (individuals assessed with the SCORE2 model) and 70 years or older (individuals assessed with the SCORE2-OP model).

The statistical analysis was performed using the data processing libraries Pandas (16), NumPy (17) and Statistics (18) to obtain insights into the overall cardiovascular risk profile and the individual risk factors underlying its calculation. For statistical inference, chi-square tests of independence and analysis of variance (ANOVA) were used for the comparison of groups, respectively for categorical and continuous variables. In the latter, Levene's test was used to test the hypothesis of equal variances and Fisher's ANOVA was replaced by Welch's ANOVA whenever this assumption was rejected (*p* < 0.05). For *post hoc* analysis of ANOVA, when the null hypothesis could be rejected, the Tukey test was employed. A significance level of 0.05 was considered for chi-square tests and ANOVA; for the Tukey test, the family-wise error rate (FWER) was maintained at a significance level of 0.05.

Given the large sample size, parametric tests were conducted even if the normality assumption was violated. The independence of observations was assumed; although there are clinical cases that justify that some individuals have their cardiovascular risk assessed more than once within a period of 7 months, it was assumed that such occurrences represented a negligible fraction of all assessments made.

Additionally, a spatial analysis was conducted to study the geographical distribution of each cardiovascular risk class, based on the geographical location of the medical users who executed the assessments in each country, throughout the entire study period. The technique used was the geolocation of internet protocol (IP) addresses, which determines the approximate geographical location of a device based on its IP address. For this purpose, databases provided by Internet Service Providers (ISPs) were used to map IP addresses to geographical locations.

The research was complemented with a comparative analysis of the adoption of the new and classic European model for cardiovascular risk assessment by the physicians. This analysis considered the proportion of assessments performed with SCORE2, SCORE2-OP, and classic SCORE models throughout a period of 17 months. The percentage (%) of non-cumulative assessments conducted using the SCORE2 and SCORE2-OP model for each month was calculated by placing in the numerator the number of assessments conducted using the SCORE2/SCORE2-OP models and in the denominator, the total number of assessments conducted in that same month with the SCORE2/SCORE2-OP models and classic SCORE model.

This work fully complies with Open Science Standards. The study design and all data used are fully described along the manuscript and on Tables and Figures.

## 3 Results

### 3.1 Sociodemographic and clinical characteristics of the evaluated individuals

During the study period (between December 2022 and July 2023), physicians conducted 24,434 cardiovascular risk assessments using the SCORE2 and SCORE2-OP models, of which approximately 60.00% were conducted on Portuguese individuals (*n* = 13,741 in Portugal, *n* = 4,620 in Italy, *n* = 5,402 in Spain and *n* = 671 in France).

As described in Table 1, 63.92% of Portuguese individuals had a total cholesterol equal to or greater than 190 mg/dl (*n* = 8,784), 18.78% were aged 70 or older (*n* = 2,581), and 24,40% had a systolic blood pressure equal to or greater than 140 mmHg (*n* = 3,352). Italy, the other country considered to have a moderate risk of cardiovascular mortality, presented a comparable proportion of individuals with total cholesterol equal to or greater than 190 mg/dl (62.92%, *n* = 2,907), but had a slightly higher proportion of elderly individuals aged 70 or older (20.61%, *n* = 952). Additionally, Italians had fewer records of systolic blood pressure equal to or greater than 140 mmHg (21.71%, *n* = 1,005).

**Table 1:** Distribution of the sociodemographic and clinical characteristics of the cardiovascular risk assessments.

	**Total**		**Portugal**		**Italy**		**Spain**		**France**	
	***n* = 24,434**		***n* = 13,741**		***n* = 4,620**		***n* = 5,402**		***n* = 671**	
Mean age in years (SD)	60 (11)		59 (11)		61 (10)		60 (10)		60 (11)	
Age in years	Percentage	*n*	Percentage	*n*	Percentage	*n*	Percentage	*n*	Percentage	*n*
40–44	9.64%	2,356	11.13%	1,529	7.77%	359	7.20%	389	11.77%	79
45–49	9.33%	2,279	11.29%	1,552	5.87%	271	7.55%	408	7.15%	48
50–54	12.69%	3,101	14.12%	1,940	7.77%	359	13.55%	732	10.43%	70
55–59	15.85%	3,873	15.56%	2,138	14.74%	681	17.48%	944	16.39%	110
60–64	17.28%	4,223	15.20%	2,089	19.37%	895	20.97%	1,133	15.80%	106
65–69	16.76%	4,094	13.91%	1,912	23.87%	1,103	17.72%	957	18.18%	122
≥70	18.45%	4,508	18.78%	2,581	20.61%	952	15.53%	839	20.27%	136
Mean systolic blood pressure in mmHg (SD)	130 (13)		131 (14)		128 (13)		128 (13)		132 (13)	
Systolic blood pressure in mmHg	Percentage	*n*	Percentage	*n*	Percentage	*n*	Percentage	*n*	Percentage	*n*
[100–110]	3.59%	878	3.94%	541	3.12%	144	3.22%	174	2.83%	19
[110–120]	11.58%	2,830	11.72%	1,611	10.80%	499	12.62%	682	5.66%	38
[120–130]	29.93%	7,312	28.60%	3,930	32.03%	1,480	32.01%	1,729	25.78%	173
[130–140]	31.52%	7,702	31.34%	4,307	32.29%	1,492	31.36%	1,694	31.15%	209
[140–150]	15.40%	3,764	15.52%	2,132	14.61%	675	14.86%	803	22.95%	154
≥150	7.97%	1,948	8.88%	1,220	7.14%	330	5.92%	320	11.62%	78
Proportion of smokers	Percentage	*n*	Percentage	*n*	Percentage	*n*	Percentage	*n*	Percentage	*n*
	17.22%	4,208	17.12%	2,353	17.27%	798	17.75%	959	14.61%	98
Mean total cholesterol in mg/dl (SD)	206 (41)		203 (40)		200 (42)		218 (40)		215 (55)	
Total cholesterol in mg/dl	Percentage	*n*	Percentage	*n*	Percentage	*n*	Percentage	*n*	Percentage	*n*
<170	18.05%	4,410	20.03%	2,752	20.13%	930	11.01%	595	19.82%	133
[170–190]	14.88%	3,636	16.05%	2,205	16.95%	783	11.07%	598	7.45%	50
[190–210]	20.73%	5,065	21.12%	2,902	23.61%	1,091	17.66%	954	17.59%	118
[210–230]	18.34%	4,480	18.73%	2,574	15.95%	737	20.07%	1,084	12.67%	85
[230–250]	13.35%	3,262	12.36%	1,698	11.30%	522	17.75%	959	12.37%	83
≥250	14.66%	3,581	11.71%	1,610	12.06%	557	22.44%	1,212	30.10%	202
Mean HDL cholesterol in mg/dl (SD)	56 (15)		54 (14)		57 (15)		59 (15)		54 (16)	
HDL cholesterol in mg/dl	Percentage	*n*	Percentage	*n*	Percentage	*n*	Percentage	*n*	Percentage	*n*
<40	10.44%	2,552	11.13%	1,673	7.77%	377	7.20%	390	11.77%	112
[40–50]	25.50%	6,230	11.29%	3,819	5.87%	1,065	7.55%	1,201	7.15%	145
[50–60]	27.76%	6,782	14.12%	3,839	7.77%	1,344	13.55%	1,422	10.43%	177
[60–70]	18.38%	4,492	15.56%	2,451	14.74%	782	17.48%	1,147	16.39%	112
≥70	17.92%	4,378	15.20%	1,959	19.37%	1,052	20.97%	1,242	15.80%	125
Mean non-HDL cholesterol in mg/dl (SD)	150 (41)		148 (39)		143 (42)		159 (40)		161 (53)	
Non-HDL cholesterol in mg/dl	Percentage	*n*	Percentage	*n*	Percentage	*n*	Percentage	*n*	Percentage	*n*
<85	5.12%	1,252	4.38%	602	8.23%	380	3.81%	206	9.54%	64
[85–100]	5.35%	1,308	5.93%	815	6.17%	285	3.05%	165	6.41%	43
[100–130]	18.90%	4,617	20.79%	2,857	21.15%	977	13.24%	715	10.13%	68
[130–150]	19.13%	4,675	19.93%	2,739	20.76%	959	16.90%	913	9.54%	64
[150–170]	20.73%	5,066	21.08%	2,896	18.40%	850	22.18%	1,198	18.18%	122
[170–190]	14.87%	3,633	13.89%	1,909	12.21%	564	19.81%	1,070	13.41%	90
[190–210]	8.86%	2,166	8.11%	1,115	7.12%	329	11.42%	617	15.65%	105
[210–230]	4.06%	993	3.30%	453	3.25%	150	6.26%	338	7.75%	52
≥230	2.96%	724	2.58%	355	2.73%	126	3.33%	180	9.39%	63

SD, standard deviation.

This proportion was close to that observed in Spain (20.78%, *n* = 1,123) but far from the proportion seen in France (34.57%, *n* = 232), two countries with low cardiovascular mortality. However, it was among Spanish that the highest proportion of assessments with total cholesterol equal to or greater than 190 mg/dl was found (77.92%, *n* = 4,209), as well as the highest proportion of assessments with an HDL cholesterol equal to or greater than 60 mg/dl (38.45%, *n* = 2,389).

A comparison of the risk assessments carried out during that period showed statistically significant differences in the mean age of individuals between Portugal and Spain, Portugal and Italy, Spain and Italy and Italy and France ([Mean ± SD]: Portugal 59 ± 11 years, Italy 61 ± 10 years, Spain 60 ± 10 years, France 60 ± 11 years; *p* < 0.01). The same assessments were performed for all the parameters. For systolic blood pressure, the results indicated statistically significant differences in individuals between Portugal and Spain, Portugal and Italy, Spain and France and Italy and France (Portugal 131 ± 14 mmHg, Italy 128 ± 13 mmHg, Spain 128 ± 13 mmHg, France 132 ± 13 mmHg; *p* < 0.01).

For total cholesterol (Portugal 203 ± 40 mg/dl, Italy 200 ± 42 mg/dl, Spain 218 ± 40 mg/dl, France 215 ± 55 mg/dl) and non-HDL cholesterol (Portugal 148 ± 39 mg/dl, Italy 143 ± 42 mg/dl, Spain 159 ± 40 mg/dl, France 161 ± 53 mg/dl), statistically significant differences were noted in individuals between Portugal and Spain, Portugal and Italy, Portugal and France, Spain and Italy and Italy and France (*p* < 0.01).

For HDL cholesterol, statistically significant differences were found in individuals between Portugal and Spain, Portugal and Italy, Spain and Italy, Spain and France, Italy and France (Portugal 54 ± 14 mg/dl, Italy 57 ± 15 mg/dl, Spain 59 ± 15 mg/dl, France 54 ± 16 mg/dl; *p* < 0.01).

No statistically significant differences were detected among the four countries for smokers, who accounted for 17.22% of all assessments (Portugal 17.12%, *n* = 2,353; Italy 17.27%, *n* = 798; Spain 17.75%, *n* = 959; France 14.61%, *n* = 98; *p* < 0.01).

Mean age at the time of evaluation using the SCORE2 model was 56 ± 8 years, approximately 19 years less than that observed in the assessments using the SCORE2-OP model (75 ± 5 years) across the four countries—Table 2. In individuals aged 70 years or older, there was a lower percentage of smokers (<70: 19.00%, *n* = 3,785; ≥70: 8.67%, *n* = 391) and lower mean levels of total cholesterol (<70: 209 ± 41 mg/dl; ≥ 70: 192 ± 41 mg/dl) and non-HDL cholesterol (<70: 153 ± 40 mg/dl; ≥70: 136 ± 40 mg/dl).

**Table 2:** Characterisation of individual risk factors by age group. For age, systolic blood pressure, and serum cholesterol, mean values and standard deviations observed were reported in each row, for the overall assessed population (total) and disaggregated for the group of individuals aged less than 70 years and those aged 70 years and older. For the “current smoker” risk factor, the percentage (%) of individuals with this characteristic were presented, first for the overall assessments, and then, for each age group.

	**Total**		**<70 years**		**≥70 years**	
	***n* = 24,434**		***n* = 19,926**		***n* = 4,508**	
Mean age in years (SD)	60 (11)		56 (8)		75 (5)	
Mean systolic blood pressure in mmHg (SD)	130 (13)		129 (13)		133 (14)	
Proportion of smokers	Percentage	*n*	Percentage	*n*	Percentage	*n*
	17.09%	4,176	19.00%	3,785	8.67%	391
Mean total cholesterol in mg/dl (SD)	206 (41)		209 (41)		192 (41)	
Mean HDL cholesterol in mg/dl (SD)	56 (15)		56 (15)		56 (15)	
Mean non-HDL cholesterol in mg/dl (SD)	150 (41)		153 (40)		136 (40)	

SD, standard deviation.

### 3.2 Cardiovascular risk profile

The SCORE2 and SCORE2-OP models showed significant differences in mean cardiovascular risk across countries over the study period (*p* < 0.01). As reported in Table 3, the probability of cardiovascular events in countries with moderate mortality risk (Portugal 6.94% ± 5.87%; Italy 7.04% ± 5.31%) was higher than that of countries with low risk (Spain 5.46% ± 3.95%; France 6.06% ± 5.02%). This conclusion holds true for both men and women, as statistical analysis did not reveal any relevant difference between genders.

**Table 3:** Cardiovascular risk percentages (by country and by cardiovascular risk class in each country) and characterisation of individual risk factors by cardiovascular risk class. For age, systolic blood pressure, and serum cholesterol, the mean values and standard deviations are reported in each row. For the “current smoker” risk factor, the percentages (%) of individuals exhibiting this characteristic in each of the indicated cardiovascular risk classes are presented in each row, first for all assessments and then for each of the four countries.

		**Low to moderate risk**		**High risk**		**Very high risk**	
10-year cardiovascular event risk in% (SD)	Mean cardiovascular event risk by country						
Breakdown							
Portugal	6.94 (5.87)	2.92 (1.42)		7.16 (2.74)		18.11 (7.47)	
Italy	7.04 (5.31)	3.33 (1.61)		7.45 (2.43)		16.98 (8.02)	
Spain	5.46 (3.95)	3.20 (1.42)		7.09 (2.49)		17.50 (5.91)	
France	6.06 (5.02)	3.16 (1.56)		7.13 (2.61)		18.71 (7.93)	
Mean age in years (SD)		54 (8)		62 (9)		73 (10)	
Breakdown							
Portugal		52 (8)		61 (10)		73 (10)	
Italy		56 (9)		64 (8)		71 (9)	
Spain		56 (8)		64 (9)		76 (10)	
France		55 (9)		63 (9)		77 (9)	
Proportion (%) of smokers		Percentage	*n*	Percentage	*n*	Percentage	*n*
		6.21%	1,517	22.78%	5,565	35.87%	8,764
Breakdown							
Portugal		5.57%	765	22.48%	3,089	31.74%	4,361
Italy		5.93%	274	18.46%	853	47.73%	2,205
Spain		7.53%	407	28.49%	1,539	40.54%	2,190
France		6.41%	43	20.42%	137	34.28%	230
Mean systolic blood pressure in mmHg (SD)		125 (12)		131 (13)		140 (16)	
Breakdown							
Portugal		126 (12)		132 (13)		140 (16)	
Italy		123 (11)		129 (12)		140 (15)	
Spain		125 (11)		132 (12)		141 (16)	
France		126 (11)		136 (12)		143 (13)	
Mean total cholesterol in mg/dl (SD)		207 (41)		206 (41)		204 (45)	
Breakdown							
Portugal		205 (38)		203 (40)		197 (44)	
Italy		198 (41)		199 (41)		213 (44)	
Spain		217 (40)		219 (41)		227 (41)	
France		205 (56)		225 (50)		227 (62)	
Mean HDL cholesterol in mg/dl (SD)		59 (15)		54 (14)		50 (13)	
Breakdown							
Portugal		58 (15)		53 (13)		50 (12)	
Italy		62 (16)		56 (14)		50 (14)	
Spain		61 (16)		54 (13)		50 (16)	
France		57 (17)		52 (14)		49 (18)	
Mean non-HDL cholesterol in mg/dl (SD)		148 (40)		152 (41)		153 (44)	
Breakdown							
Portugal		147 (37)		150 (39)		148 (43)	
Italy		136 (42)		143 (41)		162 (44)	
Spain		155 (39)		163 (39)		172 (41)	
France		148 (55)		172 (47)		178 (61)	

Legend: SD, standard deviation.

**Figure 1:**
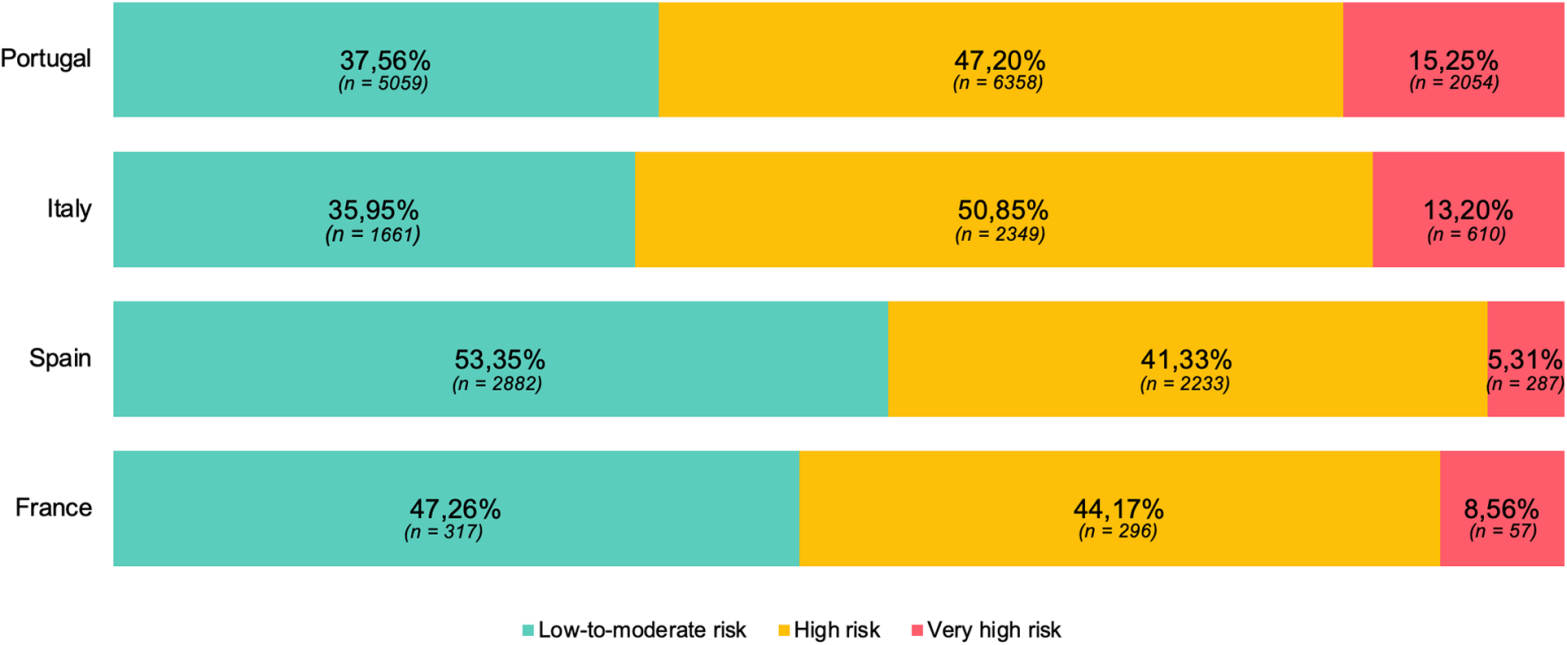
Cardiovascular risk class assessments by country, as a percentage of total volume (%). Distribution of cardiovascular risk categories, by country, obtained between December 2022 and July 2023 using the SCORE2 model for individuals aged 40–69 years and the SCORE2-OP when aged 70 and older. Percentages presented with respective absolute values (*n*). Moderate cardiovascular mortality risk: in Portugal and Italy. Low risk: Spain and France. Data collected through Tonic App, a digital mobile application for physicians.

**Figure 2:**
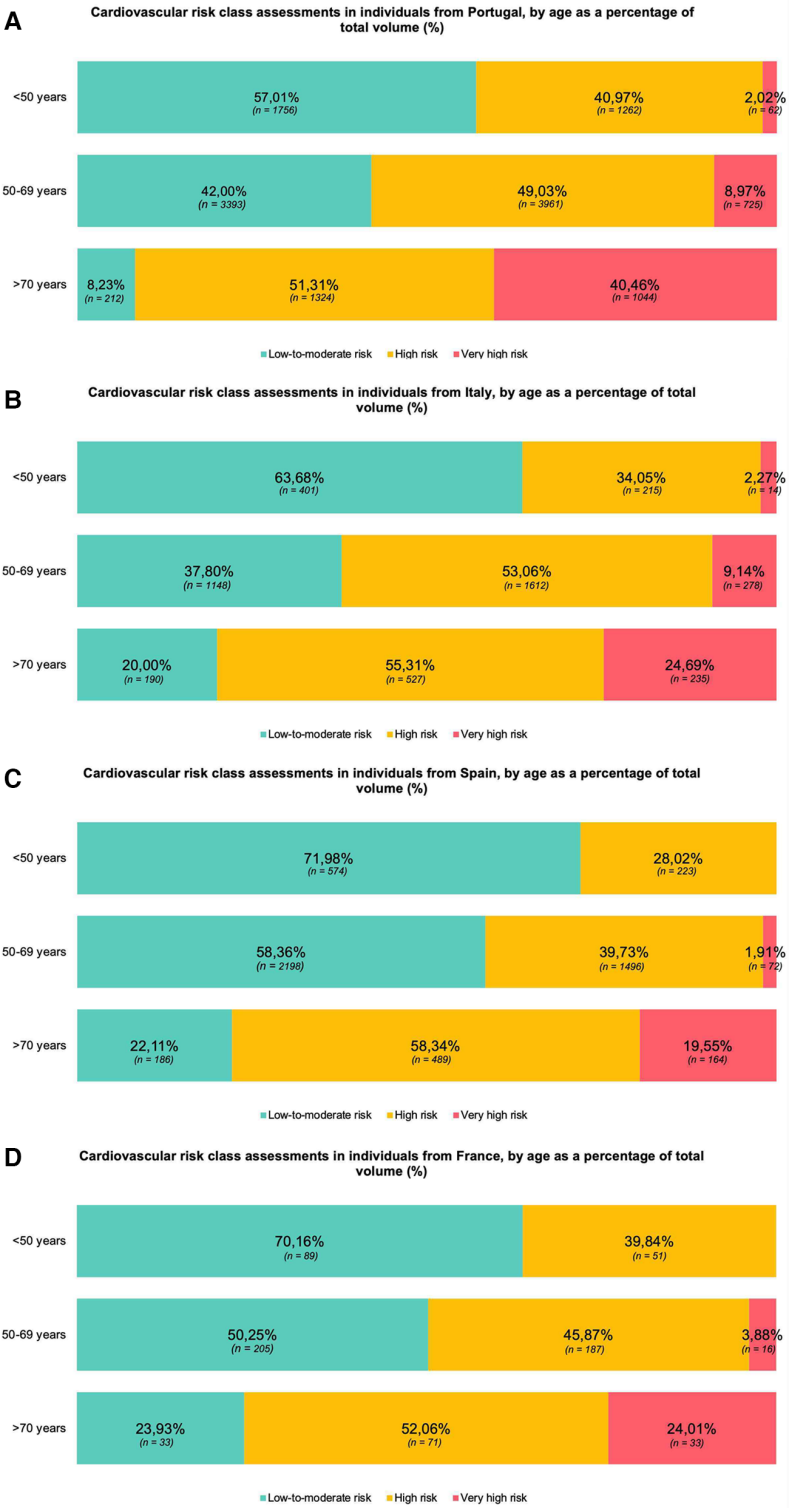
Cardiovascular risk class assessments in individuals from Portugal **(A)**, Italy **(B)**, Spain **(C)** and France **(D)**, by age as a percentage of total volume (%). Distribution of cardiovascular risk classes, by country and age group, obtained between December 2022 and July 2023 using the SCORE2 model for individuals aged 40–69 years and the SCORE2-OP when aged 70 and older. Distribution of risk classes in Portugal **(A)**, Italy **(B)**, Spain **(C)** and France **(D)**, showcasing progressively increased “high” and “very high” risk gradient from the younger to the older age group. The proportion of assessments in each risk class is presented in percentage (%). Percentages presented with respective absolute values (n). Data collected through Tonic App, a digital mobile application for physicians.

As depicted in Figure 1, approximately half of the risk estimates obtained in Spain and France (46.65%, *n* = 2,518; and 52.74%, *n* = 353; respectively) and 60% of estimates from Portugal and Italy (62.44%, *n* = 8,580; and 64.05%, *n* = 2,959; respectively) corresponded to “high” or “very high” cardiovascular risk, according to the thresholds defined in the 2021 recommendations of the European Society of Cardiology for cardiovascular risk stratification. Interestingly in the “very high” risk class (Table 3), Italy showed the lowest mean value of cardiovascular event risk (16.98% ± 8.02%), followed by Spain (17.50% ± 5.91%), Portugal (18.11% ± 7.47%) and France (18.71% ± 7.93%). In the “high” risk class, values were similar among countries whether the mortality risk was low or moderate (Portugal 7.16% ± 2.74%; Italy 7.45% ± 2.43%; Spain 7.09% ± 2.49%; France 7.13% ± 2.61%). Furthermore, the proportion of assessments in the two highest risk categories increased with age (Figure 2).

**Figure 3:**
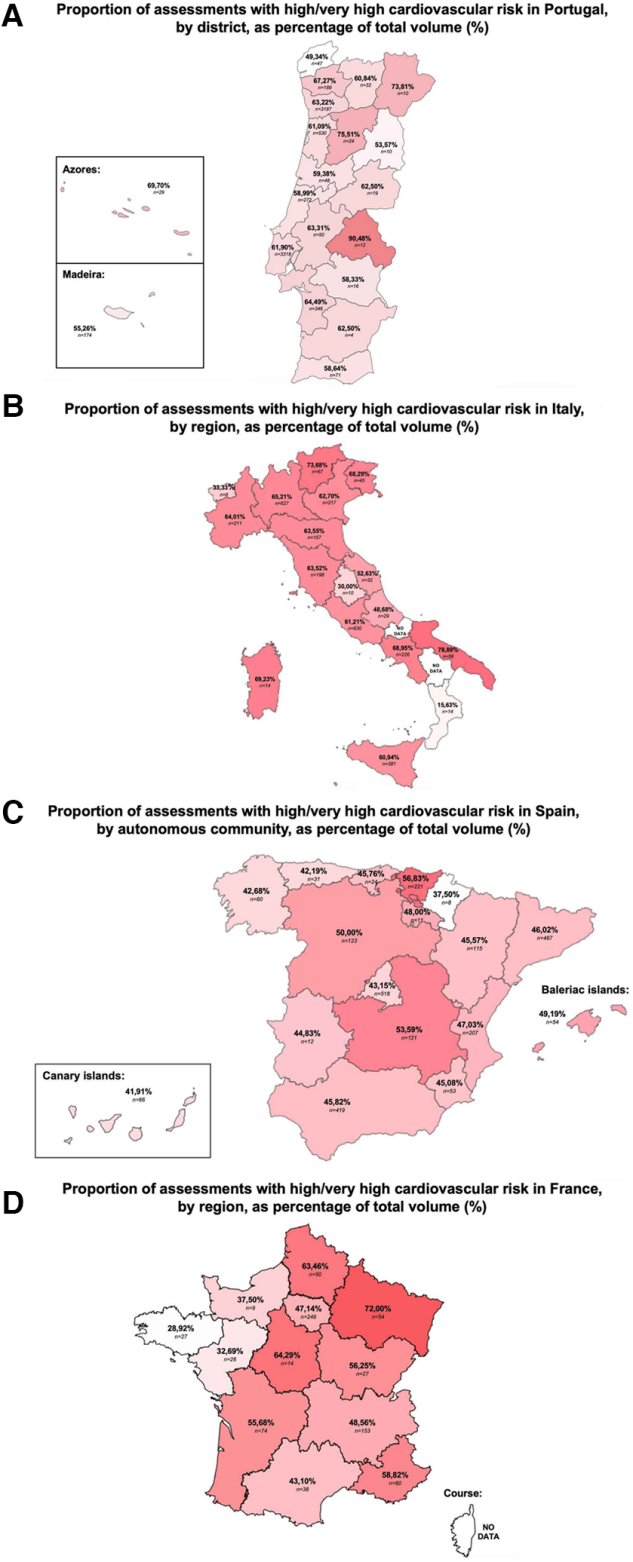
Proportion of assessments with high/very high cardiovascular risk in Portugal **(A)**, Italy **(B)**, Spain **(C)** and France **(D)**, by region, as a percentage of total volume (%). Proportion of “high” and “very high” cardiovascular risk classes, by territorial subdivision, in Portugal **(A)**, Italy **(B)**, Spain **(C)**, and France **(D)** Risk assessments were made using the SCORE2 model for individuals aged 40–69 years and the SCORE2-OP when aged 70 years and older, between December 2022 and July 2023. Substantial variations in the risk estimates classified as “high” or “very high” cardiovascular risk among the territorial subdivisions of each of the four countries were observed. Results for cardiovascular risk presented in percentage (%). Percentages presented with respective absolute values (n). Data for the Italian regions of Basilicata and Molise, as well as the French region of Corse are not presented due to a low number of cardiovascular risk assessments. Data collected through Tonic App, a mobile digital application for physicians.

The results also revealed wide variations in the risk profile within each country. Figure 3 shows that the Portuguese region of Portalegre (90.48%, *n* = 10), the Italian region of Apulia (78.89%, *n* = 56), the Basque autonomous community in Spain (56.83%, *n* = 221), and the Grand Est region of France (72.00%, *n* = 54) had the highest proportion of risk estimates classified as “high” and “very high” risk. In contrast, the lowest figures were observed in the Portuguese region of Viana do Castelo (49.34%, *n* = 47), the Italian region of Calabria (15.63%, *n* = 14), the autonomous community of Navarra in Spain (37.50%, *n* = 8), and the French region of Brittany (28.92%, *n* = 27).

### 3.3 Analysis of individual risk factors by cardiovascular risk classes

Based on the analysis of risk factors, we observed that for most individual risk factors—such as age, smoking habits, systolic blood pressure, HDL cholesterol, and non-HDL cholesterol—the values worsened as the risk category increased from “low-to-moderate” to “high” and “very high” risk classes. However, total cholesterol levels did not follow this trend. Specifically, the mean level of total cholesterol is 207 mg/dl in the low-to-moderate risk group, 206 mg/dl in the high-risk group, and 204 mg/dl in the very high-risk group (Table 3).

The sub-analysis by country (Table 3) showed that the pattern for total cholesterol was affected by the serum levels in individuals from Portugal, who made up 56.00% of the assessments in that 7-month period: Portuguese people with “very high” and “high” risk had less mean total cholesterol than those with “low-to-moderate” risk (197 ± 44 and 203 ± 40 mg/dl vs. 205 ± 38 mg/dl, respectively).

**Figure 4:**
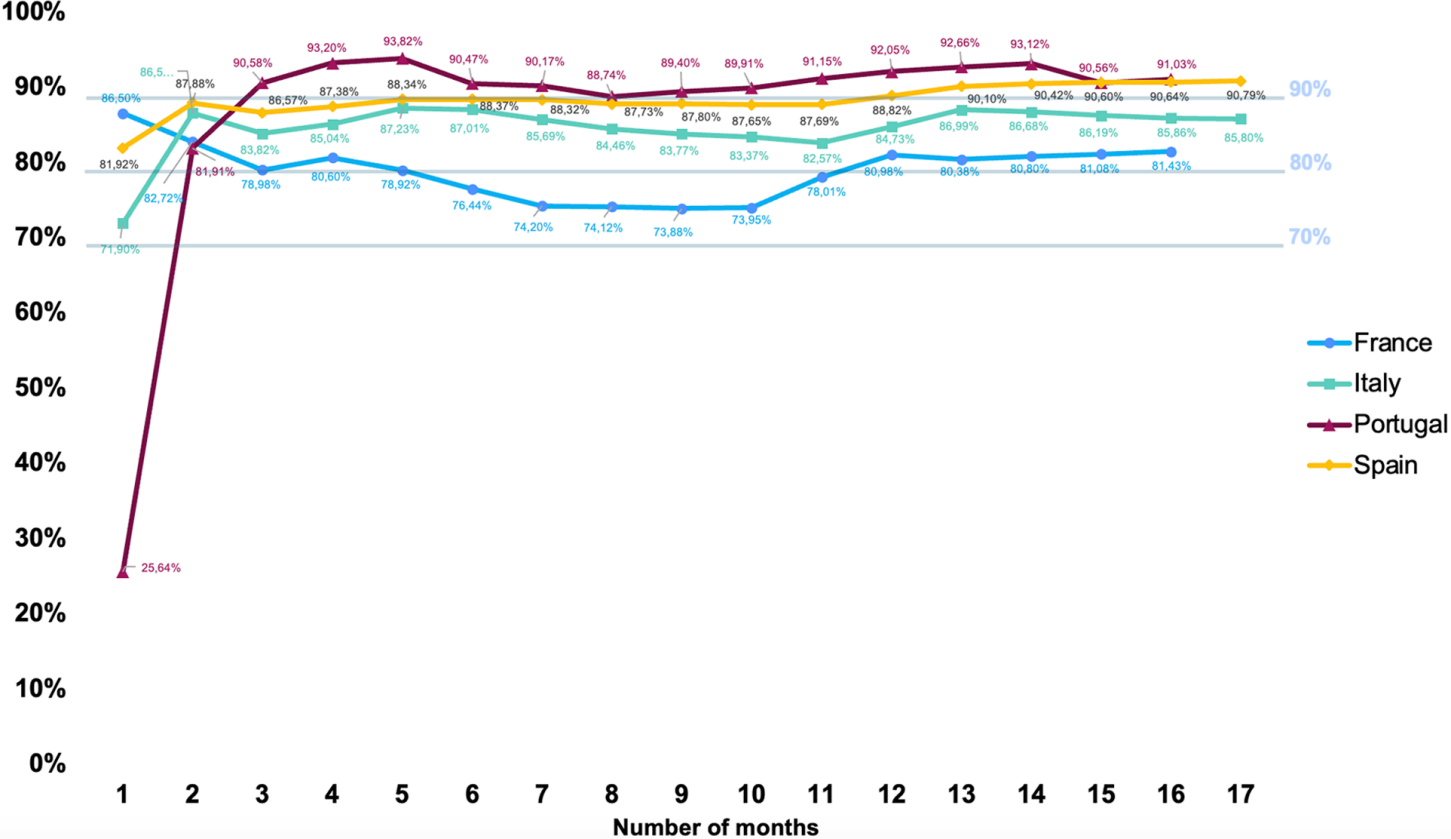
Proportion of assessments performed using the SCORE2 and SCORE-OP models, as a percentage of total volume (%). Proportion of cardiovascular risk assessments performed using SCORE2 and SCORE2-OP in comparison to the classic SCORE model, in each country, during the period that followed the introduction of the new cardiovascular risk calculation tool in Tonic App, a mobile digital application for physicians. The proportion of assessments performed with the new models oscillated between 88.73% and 93.82% in Portugal, 86.57% and 90.78%in Spain, 73.87% and 81.43%in France, and 82.56% and 87.22% in Italy. The percentage (%) represents the non-cumulative proportion of assessments conducted using the SCORE2 model and the SCORE2-OP, for each month between February 2022 and July 2023. Moderate cardiovascular mortality risk: in Portugal and Italy. Low risk: Spain and France.

The proportion of assessments performed with the SCORE2 and SCORE2-OP models, in Portugal, Italy, and Spain, reached 81.91%–87.88% of the total number of assessments performed with any of the models (new or classic) within the first couple of months after the new European cardiovascular risk models became available (Figure 4). In France, the adoption rate reached 86.50% before slightly decreasing to 82.72% by the end of the second month. Over the following months, the proportion of assessments performed with the new risk models oscillated between 88.73% and 93.82% in Portugal, 86.57% and 90.78% in Spain, 73.87% and 81.43% in France, and 82.56% and 87.22% in Italy.

**Figure 5:**
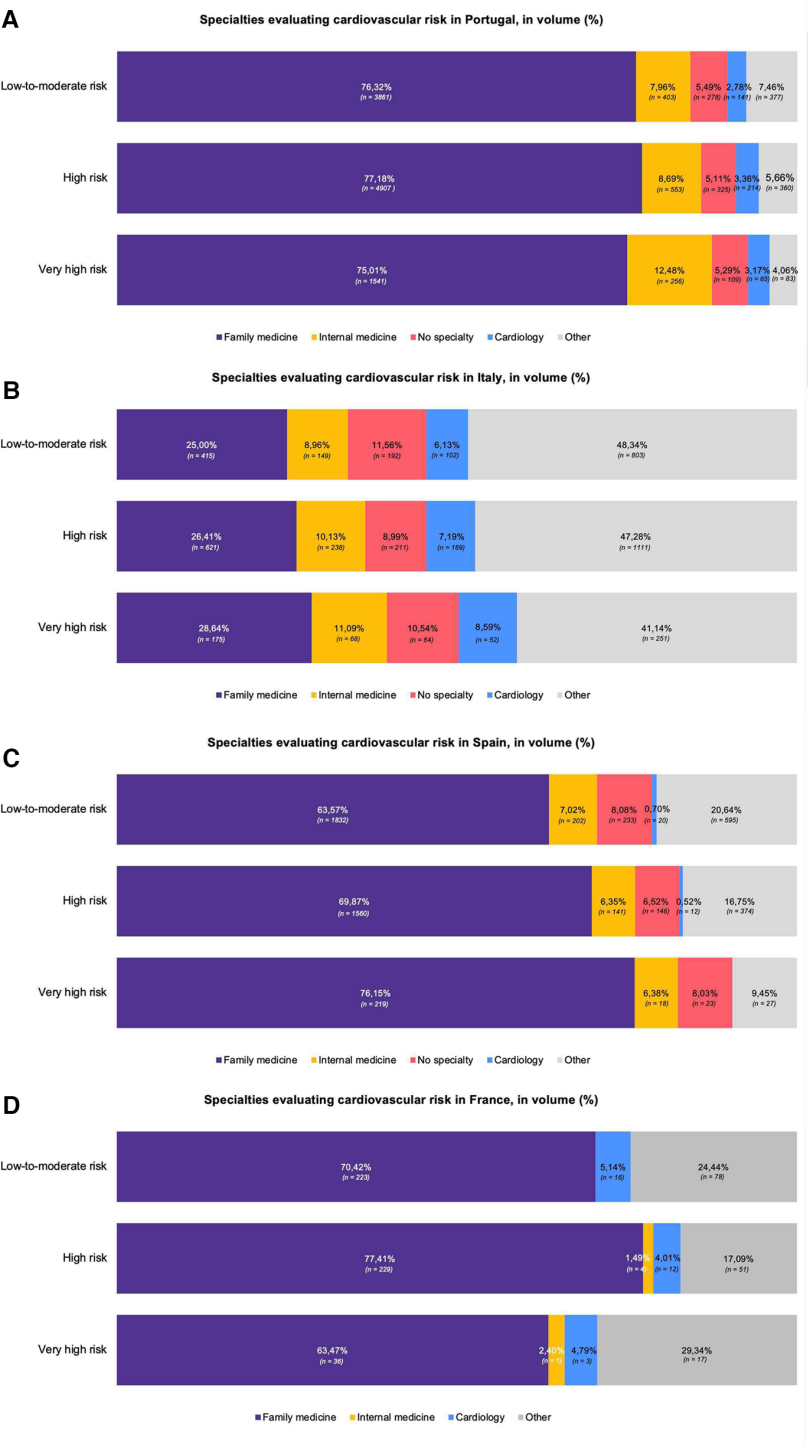
Specialties evaluating cardiovascular risk in Portugal **(A)**, Italy **(B)**, Spain **(C)** and France **(D)**, in volume (%). Distribution of cardiovascular risk assessments performed by physicians of different specialities, per country and risk class. The assessments were conducted between December 2022 and July 2023 using the SCORE2 and the SCORE2-OP models, on a mobile digital application for physicians, Tonic App. The 1st graph represents the distribution of assessments by physician speciality in Portugal **(A)**, followed by the graph for Italy **(B)**, for Spain **(C)**, and France **(D)** General practitioners are, depending on the country, responsible for one-quarter to three-quarters of the total volume of assessments in each risk class, standing out from other medical specialities. The percentage (%) corresponds to the proportion of assessments performed by physicians of each speciality and by non-specialist physicians. Percentages presented with respective absolute values (*n*).

The medical specialties that performed more assessments were general practitioners, internal medicine and cardiology in Portugal and Italy; general practitioners, internal medicine and endocrinology in Spain; and general practitioners, cardiology and geriatrics in France. As illustrated in Figure 5, general practitioners remained the speciality with the largest contribution to the risk assessments in Portugal, Spain, and France. Non-specialist doctors contributed to approximately 10% of the assessments in Spain and Italy. Internal medicine and cardiology contributed from 5.15%–20.00% of the assessments in each risk class. The contribution of other specialities was more prominent in Spain and, particularly, in Italy.

## 4 Discussion

Our real-world analysis revealed that SCORE2 and SCORE2-OP models were mostly used in the classification of high and very-high cardiovascular risk individuals. These risk categories constituted approximately half of the risk estimates obtained in individuals from Spain and France, countries with low cardiovascular mortality risk. In individuals from Portugal and Italy, countries with a moderate cardiovascular mortality risk, these risk classes accounted for nearly two-thirds of the obtained risk estimates.

In addition, our results revealed significant regional variations in cardiovascular risk across the four countries. Indeed, the areas with the highest proportion of risk estimates classified as “high” and “very high” risk were Portalegre in Portugal, Apulia in Italy, the Basque autonomous community in Spain and the Grand Est region of France. Conversely, Viana do Castelo (Portugal), Calabria (Italy), the autonomous community of Navarra (Spain) and the French region of Brittany observed the lowest risk estimates. Interestingly, the regional variations observed in France closely follow the pattern previously reported for patients who received care for cardiovascular disease among general health scheme beneficiaries (19).

Our study also highlights the interactions between individual risk factors underlying the assessment of global risk in “low-to-moderate”, “high”, or “very high risk”. These classes were associated with risk factors such as older age, smoking habits, higher systolic blood pressure, low HDL cholesterol, and high non-HDL cholesterol. Interestingly, among the evaluated Portuguese individuals, total cholesterol displayed an inverse pattern, with lower mean total cholesterol levels in those who present an increased cardiovascular risk, suggesting that the total cholesterol level is likely not be the main contributor to cardiovascular death rates in Portugal and that other factors, such as smoking habits, age and other comorbidities might be more relevant. Interestingly, a study conducted in Japan, a country with low cardiovascular mortality risk, reported a statistically significant link between low cholesterol levels and increased mortality from stroke and heart disease (20).

Knowing that the relationship between non-HDL cholesterol and cardiovascular risk is at least as strong as the relationship between LDL cholesterol and cardiovascular risk, it is therefore concerning that, in this study, only 10% of estimates classified as “high” cardiovascular risk and 4% of those in the “very high” risk class have non-HDL cholesterol below 100 and 85 mg/dl, respectively (1).

As far as we know, this is the first publication on the cardiovascular risk profile obtained, in the real world, using the SCORE2 and SCORE2-OP methods, in individuals from countries with low or moderate cardiovascular mortality risk. Although there are no other comparable studies, Csenteri et al. conducted a study with real-world data from over 85,000 individuals in Hungary, which is a country with high cardiovascular mortality risk (10). At a first glance, the findings reported in our study seem to show evidence of worst cardiovascular risk profiles (particularly in countries with moderate cardiovascular mortality risk) than those documented by Csenteri et al. in Hungary, despite the fact that this region has a higher cardiovascular mortality risk (10). Indeed, based on the numbers reported by Csenteri et al, it can be concluded that in their study the application of the SCORE2 method resulted in a classification of “high” or “very high” cardiovascular risk in 55.00% of the evaluated individuals (41.50% and 13.40%, respectively) (10). On the other hand, in our study, the “high” and “very high” cardiovascular risk profiles were found in 62.44% of patients in Portugal, 64.05% in Italy, 46.67% in Spain and 52.74% in France. However, while looking at the study designs, in Csenteri et al, the participants were aged between 40 and 65 years, while in the present study, over one-third of the assessments were performed on individuals aged 65 years or older. In fact, in our study, more than one-sixth of the assessments were performed in individuals aged 70 years or older, with a mean age of 75 years for those evaluated with SCORE2-OP. Although these individuals had lower mean levels of total cholesterol and non-HDL cholesterol, as well as a lower proportion of smokers compared to the group of individuals younger than 70 years, the proportion of individuals with “high” or “very high” risk was substantially higher in the older age group: more than 18.00%–33.00% higher, in absolute terms, compared to the proportions observed in adults aged 50–69 years. Interestingly, when we restricted our analysis to the same age group described by Csenteri et al. (40 and 65 years), the proportion of individuals with “high” or “very high” risk in our study dropped to 47.00% in Portugal, 48.01% in Italy, 33.25% in Spain, and 36.37% in France. Thus, this data suggests a more favourable risk profile in terms of mortality outcomes than that reported for Hungary, a country with higher mortality risk. Our study's broader age range likely explains the differences, as age is a primary risk factor for cardiovascular disease (1). Therefore, expanding the age range in Csenteri et al's study would likely result in a change in the cardiovascular risk profile described for Hungary, leading to a higher proportion of individuals with “high” or “very high” risk, which will likely be even more impactful and useful in the explanation of the high percentage of cardiovascular-associated deaths.

In line with this hypothesis, a smaller study with 1,317 individuals aged between 40 and 70 years in Serbia, a country with a very high cardiovascular mortality risk, revealed that 97.00% of the participants had a SCORE2 risk estimate indicating “high” or “very high” cardiovascular risk (79.00% and 18.00%, respectively) (11). Taking this into account, the risk profile found in Servia seems to be even more conducive to the very high cardiovascular mortality risk reported in the country, when compared to the reports by Csenteri et al. observed in another high mortality risk country but for a slightly younger age group, Moreover, the percentages for “high” or “very high” cardiovascular risk described in Serbia are even higher than what we observed for the exact same age group in low and moderate mortality risk regions: when we restricted our analysis to those aged 40–70 years, the proportion of individuals with “high” or “very high” risk was 54.01% in Portugal, 58.00% in Italy, 40.14% in Spain, and 45.00% in France.

Although there are no publications reporting cardiovascular risk profiles obtained using SCORE2 and SCORE2-OP in broader age ranges, a recently published study by Gavina et al, using the classic SCORE model and the corresponding risk stratification thresholds defined in the 2019 recommendations of the European Society of Cardiology, reveals that in a cohort of 78,000 individuals aged 40–80 years enrolled in the Local Health Unit of Matosinhos (Portugal), 33.00%, 2.009%, 22.00%, and 17.00% of patients had “low”, “moderate”, “high”, and “very high” cardiovascular risk, respectively (2, 7, 21) In our study, the proportion of individuals with “high” and “very high” risk is higher than what Gavina et al. found using the classic SCORE model. Thus, applying SCORE2 to the latter would lead to a higher proportion in these groups which is consistent with our findings, since: (a) the SCORE model used by Gavina et al. estimates only the risk of fatal events, while the SCORE2 model reported by us estimates the risk of both fatal and non-fatal events (7, 8); and (b) by adopting the recommendations issued in 2021 by the European Society of Cardiology for the risk stratification estimated by SCORE2, the risk thresholds applied to individuals under 50 years old are lower, allowing us to identify more individuals with “high” or “very high” cardiovascular risk at a young age (1, 21).

One of the main problems of epidemiologic research regarding the study of “high” and “very high” cardiovascular risk profiles is that physicians tend to perform risk assessments preferentially in patients with more risk factors, in detriment of patients with lower levels of major risk factors (13). We recognize that this might be a limitation of this study and that it could inflate the proportion found in the higher risk classes compared to what would be expected in a systematic assessment of risk estimation in all patients. Therefore, the proportion found needs to be confirmed in future studies, ideally prospective ones, with guarantees that the evaluated participants are representative of the population. Pseudonymisation methods that help avoid multiple cardiovascular risk assessments for the same individual could help in this pursuit. Our study has other methodologic limitations, such as the lack follow-up on clinical outcomes of the assessed individuals or the absence of information on the individuals' personal characteristics that have been reported as having a significant influence on cardiovascular risk (such socio-economic status, lifestyle choices and comorbidities, like diabetes and atheroscletotic vascular disease) (22). In addition, the lack of collection and analyses of the later information does not ensure that patient characteristics may completely represent the actual Portuguese, Italian, Spanish, and French populations, or other countries with low or moderate risk of cardiovascular mortality.

The current introduction of SCORE2 and SCORE2-OP models offers valuable insights into cardiovascular risk by incorporating both fatal and non-fatal events and incorporating age-specific risk thresholds. Nonethless, it is relevant to note that these models may lack precision for patients with multiple comorbidities or those at extreme cardiovascular risk, as they were developed for individuals who are otherwise considered healthy. Furthermore, the cut-off points for defining risk categories are not strictly evidence-based; rather, they are influenced by healthcare system constraints and economic considerations, which may not always align with individual clinical needs. Complementary models, such as the Framingham risk score, the cardiovascular quoficient score 3 (QRISK3), and atherosclerotic cardiovascular disease score (ASCVD), may offer additional risk perspectives, incorporating factors like ethnicity, socioeconomic status, and comorbidities. Integrating these alternative models could further refine cardiovascular risk stratification in diverse patient populations (10, 23).

The present study offers a preliminary insight into the risk profile of fatal and non-fatal cardiovascular disease over ten years in low and moderate cardiovascular mortality risk countries, providing a reasonably approximate portrayal of this profile in the real world and clinical practice. Indeed, our findings result from the analysis of a large number of cardiovascular risk assessments performed by nearly 7,600 physicians, who promptly adopted using SCORE2 and SCORE2-OP through a digital mobile application that has been widely implemented among the medical community. The results obtained from these analyses demonstrate internal consistency, specifically distinct risk class profiles for countries with different cardiovascular mortality risks, and more similar risk class profiles among countries with overlapping cardiovascular mortality risks. In addition, the obtained profiles align with that described in populations from countries with higher cardiovascular mortality risk and with the current paradigm of knowledge about global cardiovascular risk (e.g., the influence of age and other individual risk factors).

In this study, we presented data that highlights the relative utilisation of the SCORE2 and SCORE2-OP as physicians transitioned to the updated cardiovascular risk calculation tool provided by Tonic app. Indeed, over the experimental timeframe, the proportion of assessments performed oscillated between 88.73% and 93.82% in Portugal, 86.57% and 90.78%in Spain, 73.87% and 81.43%in France, and 82.56% and 87.22% in Italy. Overall, these high rates of adoption of the SCORE2 and SCORE2-OP models are a testament to the recognition by the medical community of the importance of cardiovascular risk assessment and the quality of the work conducted by the European Society of Cardiology in this field.

As illustrated in Figure 5, general practitioners were responsible for most of the volume of the assessments, highlighting the crucial role of primary care in the management of cardiovascular diseases. However, in Italy, the prominence of this speciality was much lower compared to the other countries. Furthermore, in Portugal, Spain and France, other medical specialities widely recognised for being involved in the management of cardiovascular risk, namely internal medicine and cardiology, were less involved in volume of risk assessments, which is worth of further research and contextualisation within the countries' healthcare landscape.

## 5 Conclusion

The profile of cardiovascular risk identified in this study revealed that in the studied countries with low-to-moderate cardiovascular mortality risk—Italy, Portugal, France and Spain—five to six out of ten individuals have a “high” or “very high” cardiovascular risk. Furthermore, this study highlights the importance of correctly mapping the cardiovascular risk profiles at regional levels in countries with different cardiovascular mortality risks. This information may be used to help health professionals target specific populations across all regions, addressing inequities and uneven cardiovascular health improvement. Our study helps reinforce the need to increase screening efforts, to develop individualised initial therapeutic approaches, and to subsequently intensify treatment based on established goals, following the recommendations issued in 2021 by the European Society of Cardiology, particularly in countries that exhibit the same risk profiles as the ones here reported. In addition, our study points towards general practitioners as being the most active professionals in the management of cardiovascular diseases, within the national health systems. Though further studies are needed to confirm this trend in other nations, this finding might prove useful for when there is a need to choose the right players to help implement successful new strategies to mitigate cardiovascular conditions.

## Data Availability

The raw data supporting the conclusions of this article will be made available by the authors, without undue reservation.
